# We cannot be “forever young,” but our children are: A multilevel intervention to sustain nursery school teachers’ resources and well-being during their long work life cycle

**DOI:** 10.1371/journal.pone.0206627

**Published:** 2018-11-01

**Authors:** Ilaria Sottimano, Gloria Guidetti, Daniela Converso, Sara Viotti

**Affiliations:** Department of Psychology, University of Turin, Turin, Italy; Indiana University, UNITED STATES

## Abstract

**Introduction:**

The aging of workers generally implies an increased number of workers with health problems or psychological diseases because of the growing distance between personal resources and job demands; the first may decrease, while the second are stable. In the preschool setting, the demands remain constant because children are always aged 0–3 years, while the preschool teacher’s personal resources decrease with age. It is, therefore, necessary to propose multilevel interventions aimed at supporting work sustainability and workers’ resources.

**Method:**

This study involved twenty-seven preschools (324 teachers with an average age of 48.7 years): the control group included seventeen schools (190 teachers with an average age of 48.5 years), five schools were assigned to experimental group one (69 teachers with an average age of 48.8), and five schools were assigned to experimental group two (65 teachers with an average age of 49.1). In this context, we proposed two protocols of multilevel intervention comprising three solutions; two of these were common to both experimental groups: psychological counseling and psychosocial intervention. The third solution differed between groups: environmental redefinition (for group one); gymnastic and vocal hygiene (for group two). We hypothesized that the interventions improve teachers’ work abilities, increase well-being, and decrease stress and burnout. Furthermore we hypothesized that there would be an improvement in the organizational climate of trust and in social job resources.

**Results:**

Data analysis showed that the interventions were effective. In particular, the experimental groups reported significant decreases in psychological exhaustion (EG2: ß = -1.48; p = .00), indolence (EG1: ß = -1.36; p = .00), and stress (EG2: ß = -0.94; p = .00). Furthermore, the experimental groups significantly increased their enthusiasm towards work (EG2: ß = 1.21; p = .01), vertical trust (EG1: ß = 0.54; p = .01), and the perception of coworker social support (EG2: ß = 0.54; p = .01). The protocol that involved the GC2 was particularly effective.

**Conclusions:**

These results demonstrate the effectiveness of the intervention in a particular job setting (preschool classroom), emphasizing the need for implementing solutions aimed at supporting workers’ well-being, especially in light of an aging workforce.

## Introduction

The aging of workforce is one of the most important shifts in industrial countries, and is a consequence of an aging population that persuaded decision-makers to raise the usual retirement age. Germany, Portugal, and Italy have the highest European age thresholds for retirement: 67 years of age or 42/43 years of contributions. For this reason, the percentage of Italian workers over 55 increased from 28.9% in 2002 to 51.9% in 2017 [1].

The literature shows that the older workers differ from their young colleagues due to biological, mental, and social characteristics that influence their needs and challenges [2]. As a result, the management of aging workers might be complicated into account that the main problem faced by the aging workforce is their decrease in personal resources despite the stability of the job demands [3]. The *ability to work* (the physical and intellectual resources on which individuals can rely to respond to emotional, cognitive, and physical demands posed by their work [4]) decreases with age [5, 6, 7, 8, 9], especially for workers involved in more physically demanding jobs [3, 4, 5, 10, 11]. However, some resources, in particular the resources of an interpersonal nature (e.g., social support and team health climate), can sustain and protect the ability to work [12, 13, 14, 15, 16, 17].

Sometimes, aging is also associated to burnout, for example, in Narumoto et al. [18] and Converso et al. [8] age predicts burnout, especially through depersonalization and emotional exhaustion. However, the supportive relationships in workplaces can make a difference for workers by potentially buffering against burnout [19, 20, 21, 22]. Workers’ perceptions of trust can also affect burnout; when trust increases, burnout decreases [23, 24].

### Public context and educational service

A very particular context within which to study aging workers is the public sector. In fact, in reason of recruitment and massive growth in public services in the 1970s and 1980s [25], the oldest workers consist primarily of public employees, such as those working in public administration [26].

Education is the sector most characterized by aging of public workers. Specifically, in Italy, the percentage of teachers over 50 years old is exceptionally high if compared to other countries [27]. The age gap between students and teachers today is very wide and will tend to increase in the future.

The preschool setting is very challenging because of its high emotional and cognitive demands in addition to the high physical demands. In this context, demands are largely constant because although children are “forever young” (i.e., between birth and 3 years old), preschool teachers are aging. Preschool teachers are often professionally satisfied because they are a significant part of the growth of very young children [28, 29], nevertheless, several studies demonstrate that working with young children requires responsibility and demands total availability of energy, attention, vigilance, sensitivity, and empathy [30, 31, 32, 33], and therefore high stress levels and burnout may occur [34, 35].

The physical demands of working with young children are very high and are an integral part of the childcare day. The preschool teachers, in their everyday work, often assume awkward postures when changing diapers or lifting children in and out of chairs and cribs. They also often sit on the floor and squat to interact with children [36, 37] (see Fig A and B in S2 File). Finally, the furniture is usually designed to be height-appropriate for children (see Fig C and D in S2 File); this often results in the preschool teacher adopting unfavorable body postures to work in this environment [38, 39, 40].

The main consequences of these high physical demands are musculoskeletal injuries: mainly neck disorders, back pain, and arm pain [28, 36, 38, 41, 42, 43]. These disorders have a significant impact on quality of life of workers, including absences due to sickness and non-fitness for work. In this way, the disorders represent a cost for the corporation that manages the service.

### Intervention to sustain aging workers

Some scholars emphasize the need for human relations (HR) practices that accommodate older workers, such as flexible work schedules, health promotion strategies, work arrangements, ergonomic solutions or lifelong learning (mentoring, job transitions, on-the-job training, adult learning, and flexible and adaptive career pathways) [3, 44, 45, 46].

However, there are few interventions specifically focused on age management and aimed at enhancing well-being throughout the entire working life cycle [47, 48] or primarily for older workers [2, 49]. For this reason, in 2016–2017, the European Agency for Safety & Health at Work (Eu-Osha) launched the campaign “Healthy Workplaces for All Ages,” with four key objectives: (a) promoting sustainable work and healthy aging from the beginning of the working life; (b) preventing health problems throughout the working life; (c) providing ways for employers and workers to manage occupational safety and health in the context of an aging workforce; and (d) encouraging the exchange of information and good practices. Through this initiative, some good business practices were promoted. The most popular and multilevel initiative before the Eu-Osha campaign was designed by BMW, which implemented several organizational changes (e.g., installing special chairs at several workstations to allow employees to work while sitting down or to relax for short periods; installing vertically adjustable tables so workstations could be adapted to each worker’s height; installing lenses to help workers distinguish small parts; introducing job rotation across workstations during a shift; hiring a physiotherapist who developed strength and stretching exercises) in order to sustain the aging workers. These changes increased productivity by 7% in 1 year, bringing the lines up to par with lines in which workers were, on average, younger [50]. More generally, the literature shows that these interventions were very useful; indeed, they decreased physical overload, reduced musculoskeletal disorders, and increased both the ability to work and employee productivity. Moreover, all participants reported a positive change in behavior, organizational climate, and mindset [50, 51].

If we focus on interventions in preschool settings, then the examples of interventions aimed on sustainability are rarer. For example, Burdorf et al. [43] reported the “ErgoKiTa project” (i.e., Ergonomics design of workplace in preschools) that aimed to ascertain the current state of knowledge available on the musculoskeletal strain of preschool teachers and to provide evaluated prevention measures. The intervention provides for the inclusion of different furniture options and individualized awareness sessions for the participant in order to highlight awkward postures adopted during the working day; however, this experience does not appear to be focused on aging.

## Material and methods

### Ethical considerations

The bioethical committee of the University of Turin, Italy, approved the study in October 2015. Research and interventions have been conducted according to the principles of the Declaration of Helsinki. Participants were recruited in accordance with the educational service management of the municipality system of Turin, Italy. Before the data collection began, a series of preparatory meetings were conducted to share the objectives and the time planned for the research with both the school administrators and the workers. The self-reporting questionnaire was administered during work hours and during a series of meetings held by a researcher of the University of Turin Department of Psychology in collaboration with the occupational physician in charge of conducting the workers’ health surveillance, as provided by the Italian law n. 81/08. After completing the questionnaire, each worker placed it in an envelope that was returned to a researcher in the Department of Psychology. During these meetings, in parallel to the self-reporting questionnaire, the occupational physician conducted some medical visits in order to evaluate the pressure of musculoskeletal disorders using the Ergonomics of Posture and Movement Questionnaire. The participants signed informed consents both for the medical examination and for the participation in experimentation.

### Study centers and design

In this paper we describe an interventional study, specifically developed for the peculiarities of the preschool teachers’ work, and on FIOH and Martin [52] indications. We proposed two protocols of multilevel interventions, comprising three solutions focused on the emotional, psychosocial, and physical support of workers. We hypothesized, more in general, that the interventions could improve the well-being at work, decrease the burnout, and sustain job resources, as evidenced by some studies [53, 54, 55, 56, 57]. More specifically, we hypothesized that the emotional support (psychological counseling) could increase the emotional health of the preschool teachers reducing burnout and stress; the psychosocial support (group intervention) could increase the perception of vertical trust and coworker support; finally that the physical support (physiotherapist and vocal therapy) could improve physical health and work ability. These hypotheses are in line with Demand-induced strain compensation model (DISC model [58]), that claims that resources can moderate the negative impact of the job demands when demands and resources match. Since preschool teachers perform tasks that are psychologically, emotionally and physically demanding, useful solutions must necessarily provide psychological and physical resources.

Our study was similar to a cluster-randomized controlled trial (CRTs) and included three groups. Two small experimental groups (EG1 and EG2) received interventions, while one control group (CG) did not.

### Participants

We included in the project the healthy preschool teachers (teachers who do not present disorders that interfere with their ability to work) who work in large institutes (i.e., more than 13 educators and more than 67 children) and in municipality preschools. The criterion for exclusion were the teachers working in private preschools or in small public preschools. We also excluded teachers with physical disorders that compromised the ability to work. The project focused on the theme of aging; however, we considered all the workers, even the few younger people at work in these settings and the remaining population aged 35–55 years.

### Procedure

In total, 27 schools were eligible for this study. The 27 schools were randomly assigned to EG1, EG2, and CG in collaboration with managers of the educational service of the City of Turin. In collaboration with the Piedmont Epidemiology service (EP) we have identified the number of participants for each group. Five preschools (including 88 teachers) constituted EG1, five preschools (including 88 teachers) constituted EG2, and 17 preschools (including 230 teachers) constituted the CG.

**Fig 1:**
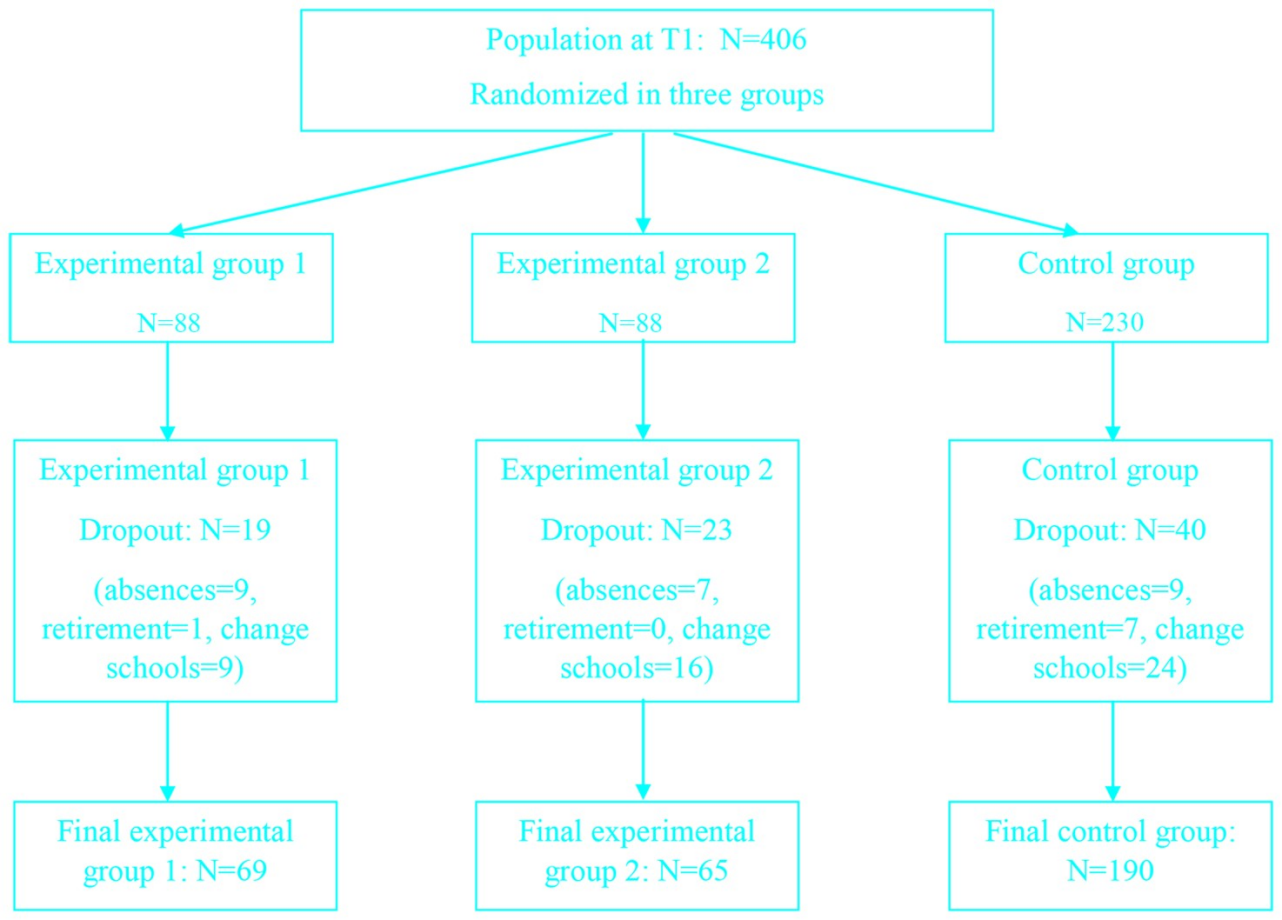
Flow diagram of the study.

During the experimentation, however, 33 participants (8.2%) dropped out at T2; the principal reasons for dropping were absenteeism (6.2%) and retirement (2%). In addition, 12.1% of the original participants were excluded because they moved to different schools; therefore, the final sample consisted of 324 participants. Of those, 69 teachers (belonging to five schools) made up EG1; 65 teachers (belonging to five schools) made up EG2, and 190 teachers (belonging to 17 schools) were included in the CG (Fig 1).

### Intervention

The protocols of interventions 1 and 2 were multilevel in an attempt to provide a coherent and adequate solution to complex health or well-being problems. Both protocols implemented three type of solutions; two of these were common to both interventions and acted on the individual and group levels.

The first activity (individual level) used psychological counseling in order to favor both the redefinition of the life and professional project (changed by postponement of retirement) and the emotional support for the preschool teachers, who were emotionally involved in care work. The preschool teachers could access three individual, face-to-face meetings, each lasting 60 minutes, with psychotherapists. The counseling space was prepared within the University environment (there was an agreement between the Municipality and the University), in order to ensure greater privacy. This activity referred to the literature on interventions of psychological support in the workplace that have shown to be very useful in promoting mental health and work ability [59].

The second activity (group level) was a worksite intervention of support to the working group. The preschool teachers met three times a psychologist trained for supporting working groups for 120 minutes after the workday. These meetings were aimed at analyzing group dynamics, improve relationships with colleagues, and group climate [49, 60].

The third solution differed between groups. In both cases the interventions were aimed at sustain educators on the physical level, but focusing for EG1 on the work environment, and for EG2 on the body.

EG1 was involved in a program of redefinition of work environment, space, and furniture, in the light of the aging workforce, as suggested by previous participatory ergonomic interventions that demonstrated to be effective for improving workers’ health [61, 62]. Preschool teachers met for three times an architect with the aim of re-define their work environments considering at the same time children needs and “aging bodies” needs. Educators were encouraged to think, draw, test new furniture and school lay out. The architect systematized and professionally drawn the different proposals (see Fig E and F in S2 File).

EG2 was involved in a program for postural gymnastic, education about movement, and education about the use of the voice. The preschool teachers met three times, for 120 minutes each time, a physiotherapist (theoretical lessons on musculoskeletal system and correct handling of weights, postural gymnastics sessions) and twice a speech therapist (theoretical lessons and vocal hygiene exercises). All meetings with the physiotherapist and speech therapist involved mixed groups of preschool teachers belonging to the 5-experimentation preschool. This solution was based on the literature about the effects of physical exercise interventions on psychosocial functioning and general well-being [63].

The protocol of intervention with all three solutions involved only the preschool teachers of two experimental groups: the preschool teachers of EG1 (NSTEG1) and the preschool teachers of EG2 (NSTEG2). The preschool teachers of the CG (NSTCG) did not receive any intervention.

### Outcome parameter

The questionnaire was specifically developed for this study and was self-administered by the participants during working time. The questionnaire included items aimed at collecting socio-demographic information (e.g., age, gender) only at the baseline measures. Furthermore, the medical examination collected health information, particularly about upper limb and rachis disorders.

The questionnaire also investigated subscales aimed at measuring study variables (i.e., work ability, stress, burnout, and job resources). Specifically, we considered the work ability, stress, and burnout as primary outcome measures in agreement with the literature that signals these dimensions in criticism referring to aging at work in highly demanding and in educational settings [3, 4, 5, 8, 10, 11]. In addition, we considered some job resources as secondary outcome measures, pursuant to literature that highlights the protective role of some work factors, especially social dimensions of work [12, 13, 14, 15, 16, 17].

*Work ability* was measured using the Work Ability Index (WAI) [4], which contains seven items used to measure self-reported work ability (α = 0.72; e.g., “Assume that your work ability at its best has a value of 10 points. How many points would you give your current work ability?”). Work ability ranged from 7 to 49 points and had four categories of responses: poor (score: 7–27); average (score: 28–36); good (score: 37–43); and excellent (score: 44–49).

We used the Spanish Burnout Inventory (SBI) [64, 65, 66, 67] to measure burnout in four dimensions:

- enthusiasm towards the job (five items, α = 0.85; e.g., “I think my job gives me positive experiences”);
- psychological exhaustion (four items, α = 0.83; e.g., “I feel emotionally exhausted”);
- indolence (six items, α = 0.69; e.g., “I don’t like taking care of some students”); and
- guilt (five items, α = 0.81; e.g., “I feel guilty about some of my attitudes at work”).

Responses on two subscales of SBI were given on a 5-point scale with a range from 0 = never to 4 = frequently. Scores for enthusiasm towards the job ranged from 0 to 20; psychological exhaustion scores ranged from 0 to 16; indolence ranged from 0 to 24; and guilt ranged from 0 to 20.

*Stress* and *Vertical Trust* were measured with the Copenhagen Psychosocial Questionnaire, short version (COPSOQ II) [68], which contains two items for stress (α = 0.83; e.g., “How often have you been stressed?”) and two items for vertical trust (α = 0.75; e.g., “Does the management trust the employees to do their work well?”). Responses on subscales were given on a 5-point scale with a range from 0 = not at all to 4 = all the time. Stress and vertical trust scores ranged from 0 to 8.

*Coworker social support* was measured with the Job Content Questionnaire (JCQ) [69], which contains six items that investigate the support from colleagues (α = 0.69; e.g., “People I work with are competent in doing their jobs?”). Responses on subscales were given on a 4-point scale with a range of 1 = strongly disagree to 4 = strongly agree. Scores ranged from 6 to 24.

We considered *age* as a control variable.

### Analyses

Baseline difference in socio-demographic variables between participant and nonparticipants, and between the EG1, EG2, and CG, were investigated using *t*-test, ANOVA, and chi-square tests. Pearson’s correlations were performed to assess the associations between the variables considered (Table 1).

**Table 1:** Correlations between variables.

	Age	Enthusiasm	Exhaustion	Indolence	Guilt	Work ability	Stress	Vertical trust	Coworker support
Age	1	-.092	.106	.046	.097	-.168**	-.018	-.068	-.083
Enthusiasm at work	-.092	1	-.248**	-.218**	-.191**	.192**	-.260**	.069	.194**
Psychological exhaustion	.106	-.248**	1	.379**	.337**	-.307**	.661**	-.197**	-.076
Indolence	.046	-.218**	.379**	1	.415**	-.197**	.378**	-.268**	-.061
Guilt	.097	-.191**	.337**	.415**	1	-.158**	.280**	-.050	-.039
Work ability	-.168**	.192**	-.307**	-.197**	-.158**	1	-.281**	.141*	-.026
Stress	-.018	-.260**	.661**	.378**	.280**	-.281**	1	-.203**	-.128*
Vertical trust	-.068	.069	-.197**	-.268**	-.050	.141*	-.203**	1	.019
Coworker social support	-.083	.194**	-.076	-.061	-.039	-.026	-.128*	.019	1

* Correlation is significant at the 0.05 level

** Correlation is significant at the 0.01 level

To evaluate the effects of the interventions and to control for the effects of single preschool belonging, we adopted the linear mixed model. This approach has several advantages over traditional analyses using a linear model (e.g., ANOVA). First of all, the clustering effect of the preschool belonging can be considered in the analyses.

For the linear mixed model, the fix factors were: main effects of group (EG1, EG2, CG), main effects of time (T1 or baseline and T2), and interaction between groups and time. The random part of the model consisted of the preschool and the individual effects within the preschools. The random part is required to account for the clustering effects of preschools (on individuals) and of individuals (on repeated measurements), in order to obtain significance tests that correctly reflect features of our design. Age of participants was used as a control variable.

The estimation and significance testing was carried out by using Mixed Models of the Statistical Package for Social Sciences software (SPSS) version 24. The intraclass correlation coefficient (ICC) was estimated to measure the clustering effect and the proportion of variance tied to clustering.

## Results

ICC shows that the proportion of variance in work ability between preschools was 6.1%. Regarding burnout, the proportion of variance between preschools were: enthusiasm (3.5%), exhaustion (9.4%), indolence (3%), and guilt (5.7%). The proportion of variance in stress between preschools was 8.4%. As to job resources, the proportion of variance between preschools in vertical trust was 4.9%, and variance in coworkers’ social support was 3.8%.

### Dropout analysis

Dropout analyses show no difference between the dropout group and the final group (see Tables A, B, and C in S1 File). The *t*-test shows that there were no differences based on age for the CG or for experimental groups (EG1, age: *t*(23) = 0.70, *p* = .49; EG2, age: *t*(29) = 1.15, *p* = .26; CG, age: *t*(51) = 0.17, *p* = .87). Moreover, with respect to the main musculoskeletal pains reported, there were no differences between dropout group and the final group for CG and the experimental groups. To EG1, shoulder pain: χ^2^ 1, (N = 88) = 0.40, *p* = .53; cervical pain: χ^2^ 1, (N = 88) = 1.00, *p* = .32; and low back pain: χ^2^ 1, (N = 88) = 0.34, *p* = .56. To EG2, shoulder pain: χ^2^ 1, (N = 88) = 0.48, *p* = .49; cervical pain: χ^2^ 1, (N = 88) = 0.32, *p* = .57; and low back pain: χ^2^ 1, (N = 88) = 0.03, *p* = .87. To CG, shoulder pain: χ^2^ 1, (N = 230) = 1.46, *p* = .23; cervical pain: χ^2^ 1, (N = 230) = 0.01, *p* = .95; and low back pain: χ^2^ 1, (N = 230) = 1.52, *p* = .22.

Regarding the major study variable, there were no differences between the dropout group and the final group for CG and experimental groups. To EG1, enthusiasm: *t*(81) = -1.38, *p* = .17; psychological exhaustion: *t*(83) = 0.71, *p* = .48; indolence: *t*(83) = 1.50, *p* = .14; guilt: *t*(82) = -0.32, *p* = .75; work ability: *t*(84) = 1.23, *p* = .22; stress: *t*(82) = -0.47, *p* = .64; vertical trust: *t*(78) = 0.13, *p* = .90; and coworker social support: *t*(80) = -0.52, *p* = .61. To EG2, enthusiasm: *t*(24) = 0.81, *p* = .43; psychological exhaustion: *t*(27) = 0.73, *p* = .47; indolence: *t*(81) = 0.02, *p* = .98; guilt: *t*(79) = -1.14, *p* = .26; work ability: *t*(29) = 1.99, *p* = .06; stress: *t*(85) = -0.16, *p* = .87; vertical trust: *t*(79) = 0.20, *p* = .84; and coworker social support: *t*(77) = 0.81, *p* = .42. To CG, enthusiasm: *t*(39) = 0.75, *p* = .45; psychological exhaustion: *t*(223) = -1.47, *p* = .14; indolence: *t*(219) = -0.42, *p* = .67; guilt: *t*(221) = -0.25, *p* = .80; work ability: *t*(226) = 0.80, *p* = .42; stress: *t*(225) = -0.17, *p* = .87; vertical trust: *t*(210) = 0.37, *p* = .71; and coworker social support: *t*(219) = 1.70, *p* = .09.

### Analysis at T1

At the first time (T1), the 324 preschool teachers ranged from 20 to 63 years old (Table 2: mean EG1: 48.8 years, SD 6.6; mean EG2: 49.1 years, SD 6.4; and CG: 48.5 years, SD 7.7). Among all the teachers, 99% were women (100% in EG1, 100% in EG2, and 98.9% in CG), and 1% were male (0% in EG1, 0% in EG2, and 1.1% in CG). The main musculoskeletal pains detected by occupational physician were shoulder pain (44.9% in EG1, 43.1% in EG2, and 27.9% in CG), cervical pain (55.1% in EG1, 58.5% in EG2, and 69.5% in CG) and low back pain (65.2% in EG1, 67.7% in EG2, and 70.0% in CG).

**Table 2:** Baseline characteristics of the experimental and control groups (mean and standard deviations).

	Experimental Groups 1 (n = 69)		Experimental Groups 2 (n = 65)		Control Group (n = 190)		ANOVA	
	M	DS	M	DS	M	DS	F	*p*
Age	48.83	6.65	49.11	6.45	48.49	7.73	0.19	.82
Enthusiasm at work	14.29	3.04	14.28	3.00	15.22	3.13	3.37	.04
Psychological exhaustion	7.75	2.96	8.05	3.19	7.06	3.26	2.76	.06
Indolence	4.65	2.91	4.61	3.18	3.80	2.47	2.96	.05
Guilt	4.44	3.42	4.05	2.59	3.76	2.73	1.29	.28
Work ability	33.44	6.96	33.88	6.10	33.26	6.00	0.23	79
Stress	3.19	1.66	3.69	1.85	3.16	1.94	2.05	.13
Vertical trust	3.29	1.45	3.43	1.40	3.69	1.36	2.76	.10
Coworker social support	18.38	2.21	17.51	2.18	18.4	1.71	5.08	.01

There were no difference between the three groups regarding age (*F*(320) = 0.19, *p* = .82), gender (χ^2^ 1, (N = 324) = 1.42, *p* = .49), and musculoskeletal disorders (cervical pain: χ^2^ 1, (N = 324) = 5.73, *p* = .06; and low back pain: χ^2^ 1, (N = 324) = 0.56, *p* = .75). There was, however, a difference between the three groups as to shoulder pain, which was more widespread in the two experimental groups (χ^2^ 1, (N = 324) = 9.09, *p* = .01).

Table 2 shows that there were no differences between the three groups about psychological exhaustion (*F*(315) = 2.76, p = .06), indolence (*F*(169) = 2.96, p>.05), guilt (*F*(169) = 1.29, p = .28), work ability (*F*(321) = 0.23, p = .79), stress (*F*(321) = 2.05, p = .13), and about vertical trust (*F*(300) = 2.76, p = .10). There was, however, a difference between the three groups about enthusiasm towards work (*F*(307) = 3.37, p = .04) and coworkers’ social support (*F*(305) = 5.08 p = .01).

### Intervention effects on enthusiasm towards work, psychological exhaustion, indolence, guilt, work ability and stress

**Fig 2:**
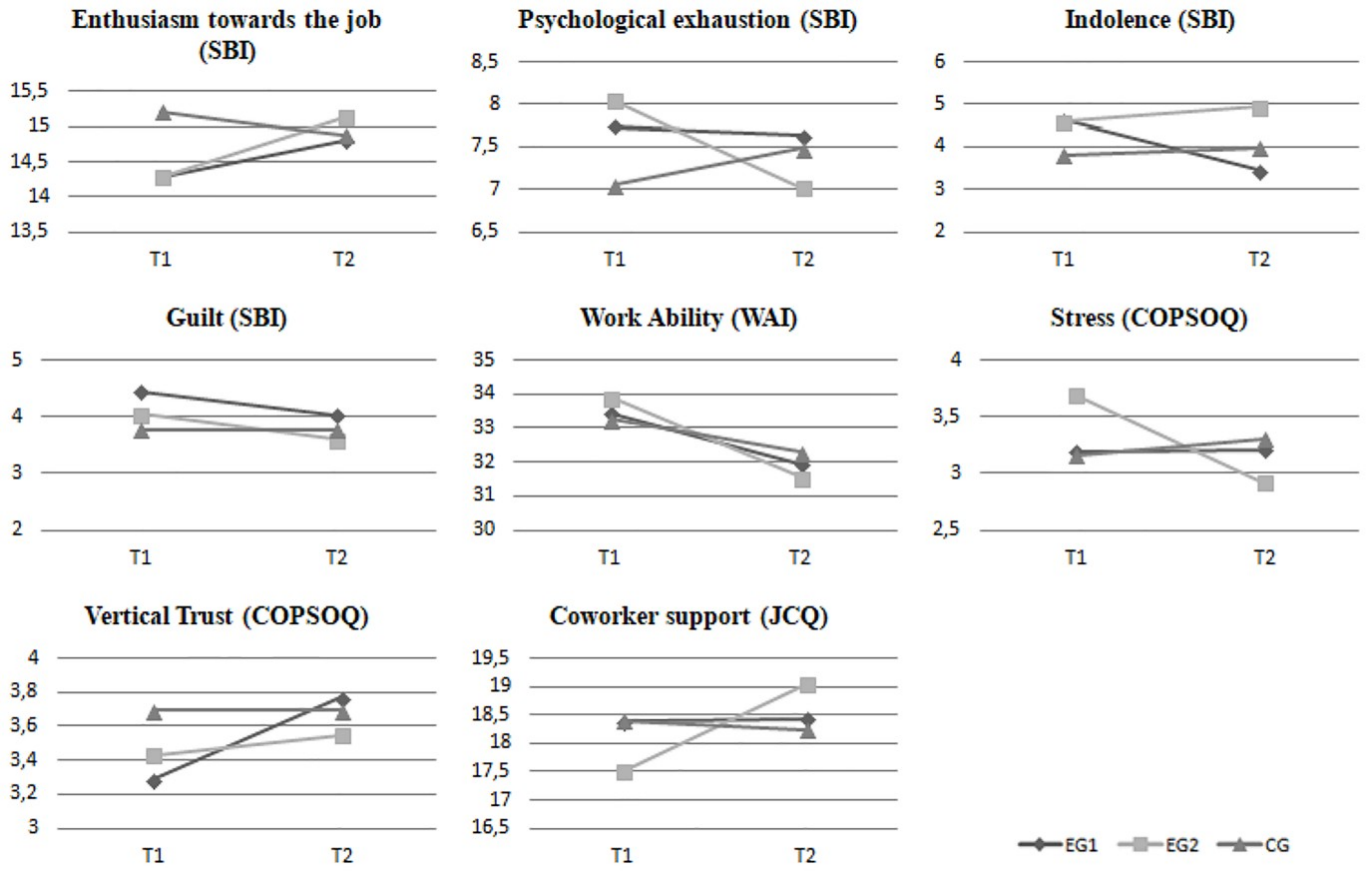
Outcome variables during experimentation 1, experimentation 2 and no-intervention.

Fig 2 shows that, from T1 to T2, the mean value of enthusiasm towards work improved for EG1 and EG2 and decreased for CG. The intervention resulted in a significant effect on enthusiasm towards work because there was an interaction effect between group and time. Specifically, there was a significant interaction effect between EG2 and time (ß = 1.21; p = .01). This means that, when compared to the CG, the EG2 significantly increased enthusiasm towards work.

Again, from T1 to T2, the mean value of psychological exhaustion, decreased for EG1 and EG2 and increased for CG. The intervention resulted in a significant effect on psychological exhaustion because there was an interaction effect between group and time. Specifically, there was a significant interaction effect between EG2 and time (ß = -1.48; p = .00). This means that, when compared to the CG, the EG2 significantly decreased their level of psychological exhaustion.

Fig 2 shows that from T1 to T2, the mean value of indolence decreased for EG1 and increased for EG2 and CG. The intervention resulted in a significant effect on indolence because there was an interaction effect between group and time. Specifically, there was a significant interaction effect between EG1 and time (ß = -1.36; p = .00). This means that, when compared to the CG, the EG1 significantly decreased in indolence.

From T1 to T2, the mean value of guilt decreased for EG1 and EG2 while it remained stable for CG. The intervention, however, did not have a significant impact on the reduction of guilt; in fact, there was no significant interaction effect between group and time.

The work ability, conversely, decreased for all groups. There was no significant interaction effect between group and time.

Fig 2 shows again that, from T1 to T2, the mean value of stress decreased for EG2, it remained stable for EG1, and it increased for CG. The intervention resulted in a significant effect on stress because there was an interaction effect between group and time. Specifically, there was a significant interaction effect between EG2 and time (ß = -0.94; p = .00). This means that, when compared to the CG, there was significantly decreased stress in the EG2.

### Intervention effects on vertical trust and coworker social support

From T1 to T2, the mean value of vertical trust increased for EG1 and EG2 and remained stable for CG. The intervention resulted in a significant effect on vertical trust because there was an interaction effect between group and time. Specifically, there was a significant interaction effect between EG1 and time (ß = 0.54; p = .01).

Finally, the mean value of coworker social support increased for EG1 and EG2 and decreased for CG, from T1 to T2 (Fig 2). The intervention resulted in a significant effect on coworkers’ support because there was an interaction effect between group and time. Specifically, there was a significant interaction effect between EG2 and time (ß = 0.54; p = .01).

### Intervention effects on work ability and psychological exhaustion

At T1, there emerged a mediation effect of work ability between age and psychological exhaustion, both for the experimental group (considered both) and CG. An indirect effect of age on psychological exhaustion for the experimental group was: *b* = .022, BCa CI(.002, .061); the indirect effect of age on psychological exhaustion for the CG was: *b* = .022, BCa CI (.003, .050). At T2, this indirect effect was present only in the CG, and it was not present in the experimental group. For the CG: *b* = .047, BCa CI (.018, .088); for the experimental group: *b* = .015, BCa CI (-.003, .046).

## Discussion

Data analysis show that between the preschool teachers of experimental groups (NSTEG) burnout, stress, vertical trust and coworker support significantly decreased.

In particular, in NSTEG1 indolence decreased, while in NSTEG2 psychological exhaustion decreased and enthusiasm towards work increased. These results are consistent with those studies that highlighted a relationship between workplace intervention—especially participatory intervention—and burnout [70, 71, 72]. This is also true of our solution of redefinition of work environment (that involved NSTEG1) and the psychosocial intervention with a working group (that involved both NSTEG1 and NSTEG2). Furthermore, Sjögren et al. [63] and Naczenski et al. [73] show that physical exercise intervention has an impact on burnout; accordingly, we could conclude that the physiotherapy training for NSTEG2 also could affect burnout levels by decreasing exhaustion.

Our study also highlights the effectiveness of the intervention on stress, in particular for the NSTEG2. These findings are in accord with those of Mikkelsen and Gundersen [74] and Arnetz et al. [75]: there is a relationship between participatory organizational intervention and positive changes in job stress.

More generally, these results support Hargrove et al. [76], Kooji et al. [44], De Lange et al. [45], Martin el al. [52] and the FIOH, which all emphasize the importance of acting on multiple levels to support psychological health at work and, in particular, the working ability of aging staff. Our study also stresses the efficacy of interventions on job resources—in particular, those of an interpersonal nature—that could moderate the negative effect of aging on work ability and protect the preschool teachers from burnout [12, 14, 15,77, 78]. In fact, from T1 to T2, NSTEG 1 and NSTEG 2 significantly improved in vertical trust and coworker social support compared to the NSTCG. Our findings revealed that after the multilevel interventions, there were significantly improved perceptions of social resources in the work context, whereas no improvement was observed in the NSTCG at the same time points. These findings are consistent with Hätinen et al. [71], Hargrove et al. [76], Pignata and Winefield [79] and Martin et al. [52], which held that the intervention that involves workers and work groups has an impact on organizational climate and social support. Finally, the disappearance of the mediation effect on the WAI between age and psychological exhaustion for NSTEG may indicate that the intervention itself did not have a direct impact on work ability. Instead, the intervention weakened this indirect relationship, containing the adverse effects of the decline of work ability in psychological exhaustion.

Our results can be explained, in the light of the DISC model and of the occupational psychology perspective [58, 80], as the consequences of the resources-demands match at the three intervention levels (individual, group, organization). The psychological counseling meetings (intervention that acts on the individual level, based on resources of a psychological kind) supported the teachers who participated in the experimentation to better mentalize their work, cope with the emotionally aspects and revitalize their motivation to work, and therefore reduced their stress and burnout. The group meetings (intervention that acts on the group level, based on resources of a psychological kind) supported work and personal relationships within colleagues, and therefore improve their perception of social support and organizational trust.

The physiotherapy and speech therapy (intervention that acts on the individual level, based on resources of a physical kind) was expected to enhance work ability and ameliorate physical health, but this did not happen. Even if the decreased psychological exhaustion indicate a positive result for psychophysical health, physical demands are probably too high and/or the physical resources provided too low to obtain a major, positive change.

Finally the intervention of redefinition of work environment, space, and furniture (intervention that acts on the group level, based on resources of a physical kind) strengthened the sense of belonging to the school (as a building, as a work group) and enhanced involving, therefore increasing the feeling of trust in the organization. As for the previous intervention, physical demands are probably too high and/or the physical resources provided too low to obtain a major, positive change on the physical side. Moreover, changes in schools lay out and the introduction of new furniture, need a longer time to verify their impact on health and work ability.

In conclusion, these results demonstrate the effectiveness of interventions in a particular job setting (i.e., a preschool), and emphasize the need for implementing solutions aimed at supporting workers’ well-being, especially in light of an aging workforce that could compromise work ability and increase levels of stress and burnout. This is even more relevant in settings such as preschools, where workers are exposed to physical, cognitive, and emotional demands at the same time. It should be remembered, however, that there were no direct effects on work ability, which instead worsened for everyone. This data could confirm the critical issues—already widely reported in the literature—regarding the negative impact of physically demanding tasks on work ability within the life cycle [3, 4, 5, 10, 11].

### Limitations and suggestion for further studies

Typically, the literature identify some limits in organizational interventions such as: the lack of control group, the inability to randomly assign people to groups experiments, the reduced sample size, the lack of follow-up and the exclusive use of subjective measures and self-report. Often the ‘good practices’ report solutions that are not always associated with an impact measurement and an ex ante evaluation. In this direction, our study has some strong points that exceed traditional limits, for example, there is a control group, the sample size is quite large compared to other studies, at least for the Italian context; it is a multilevel intervention and we tried to follow a methodology as close as possible to clinical trials. Finally, our study involves a particular context: the preschool environment and the public sector, characterized by a generally higher average age and scarcity of resources.

Nevertheless, some limits exist, including the lack of a second follow-up. Furthermore, the interventions did not affect work ability.

Future interventions of this kind should consider a follow-up study at 6 months and 1 year after T2, to check the stability of the results.

## Supporting information

S1 File Dropout analyses. Dropout analyses for experimental group 1, 2 and control group.
(DOCX)

S2 File Images of the experimentation. Harmful positions abstained by the preschool teachers and furniture in preschool context.
(DOCX)
