# Supplementary material for: We cannot be “forever young,” but our children are: A multilevel intervention to sustain nursery school teachers’ resources and well-being during their long work life cycle
Source: PLoS One. 2018 Nov 1;13(11):e0206627. doi: 10.1371/journal.pone.0206627 (PMC6211713; doi:10.1371/journal.pone.0206627)
Supplement: S1 File — Dropout analyses for experimental group 1, 2 and control group. (DOCX) [file pone.0206627.s001.docx]

**Supplemental Tables S1. Dropout analyses**

**Table A S1**. Dropout analyses for experimental group 1 (mean and standard deviations for baseline characteristics)

|  | Dropout (n=19) | | Experimental group 1 (n=69) | |
| --- | --- | --- | --- | --- |
|  | M | DS | M | DS |
| Age | 47.21 | 9.44 | 48.83 | 6.65 |
| Enthusiasm at work | 15.39 | 2.77 | 14.29 | 3.04 |
| Psychological exhaustion | 7.17 | 3.42 | 7.75 | 2.96 |
| Indolence | 3.58 | 2.01 | 4.65 | 2.91 |
| Guilt | 4.72 | 3.16 | 4.44 | 3.42 |
| Work ability | 31.05 | 8.61 | 33.44 | 6.96 |
| Stress | 3.41 | 1.91 | 3.19 | 1.66 |
| Vertical trust | 3.24 | 1.30 | 3.29 | 1.45 |
| Coworker social support | 18.71 | 2.52 | 18.38 | 2.21 |

**Table B S1**. Dropout analyses for experimental group 2 (mean and standard deviations for baseline characteristics)

|  | Dropout (n=23) | | Experimental group 2 (n=65) | |
| --- | --- | --- | --- | --- |
|  | M | DS | M | DS |
| Age | 46.57 | 9.91 | 49.11 | 6.45 |
| Enthusiasm at work | 13.30 | 5.17 | 14.28 | 3.00 |
| Psychological exhaustion | 7.29 | 4.42 | 8.05 | 3.19 |
| Indolence | 4.59 | 2.99 | 4.61 | 3.18 |
| Guilt | 4.90 | 3.65 | 4.05 | 2.59 |
| Work ability | 29.87 | 8.94 | 33.88 | 6.08 |
| Stress | 3.77 | 2.45 | 3.69 | 1.85 |
| Vertical trust | 3.50 | 1.43 | 3.43 | 1.40 |
| Coworker social support | 17.05 | 2.16 | 17.51 | 2.18 |

**Table C S1**. Dropout analyses for control group (mean and standard deviations for baseline characteristics)

|  | Dropout (n=40) | | Control group (n=190) | |
| --- | --- | --- | --- | --- |
|  | M | DS | M | DS |
| Age | 48.23 | 9.19 | 48.49 | 7.73 |
| Enthusiasm at work | 15.57 | 4.88 | 15.22 | 3.13 |
| Psychological exhaustion | 7.95 | 4.03 | 7.06 | 3.26 |
| Indolence | 4.00 | 3.27 | 3.80 | 2.47 |
| Guilt | 3.89 | 3.18 | 3.76 | 2.73 |
| Work ability | 32.41 | 6.26 | 33.26 | 6.00 |
| Stress | 3.22 | 1.97 | 3.16 | 1.94 |
| Vertical trust | 3.60 | 1.50 | 3.69 | 1.36 |
| Coworker social support | 17.85 | 2.40 | 18.40 | 1.71 |
