# Supplementary material for: We cannot be “forever young,” but our children are: A multilevel intervention to sustain nursery school teachers’ resources and well-being during their long work life cycle
Source: PLoS One. 2018 Nov 1;13(11):e0206627. doi: 10.1371/journal.pone.0206627 (PMC6211713; doi:10.1371/journal.pone.0206627)

**Supporting information S2. Images of the experimentation**

**S2 Fig A: harmful positions abstained by the preschool teachers**


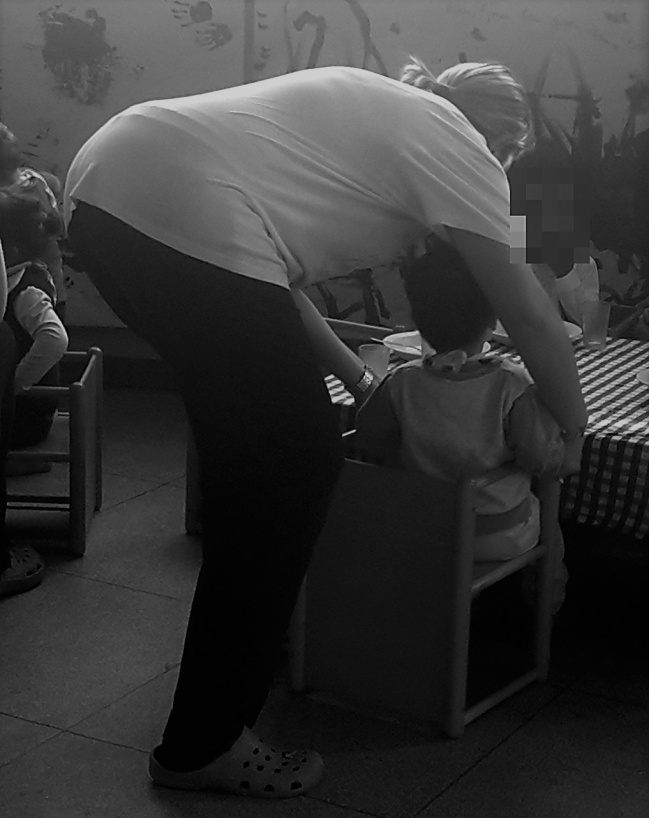


**S2 Fig B: harmful positions abstained by the preschool teachers**


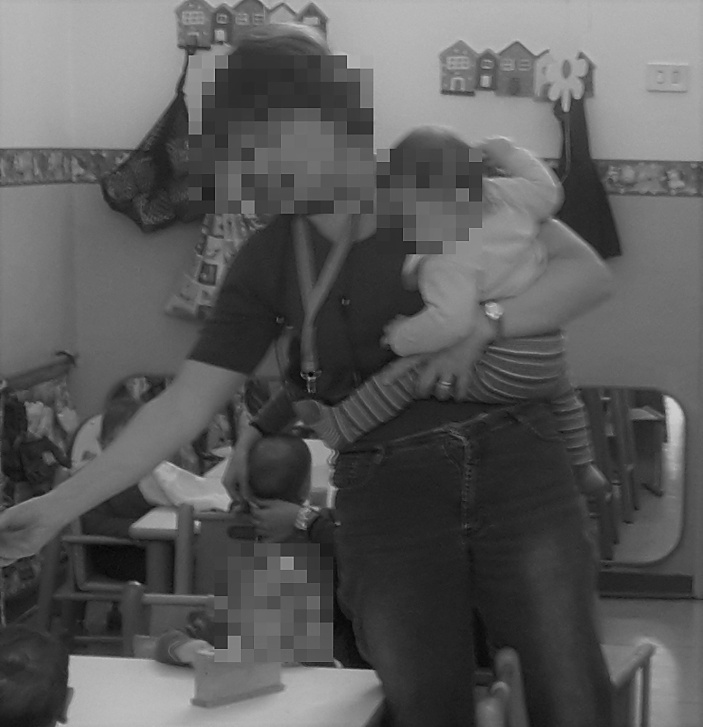


**S2 Fig C: furniture and preschool context (sleepy room)**


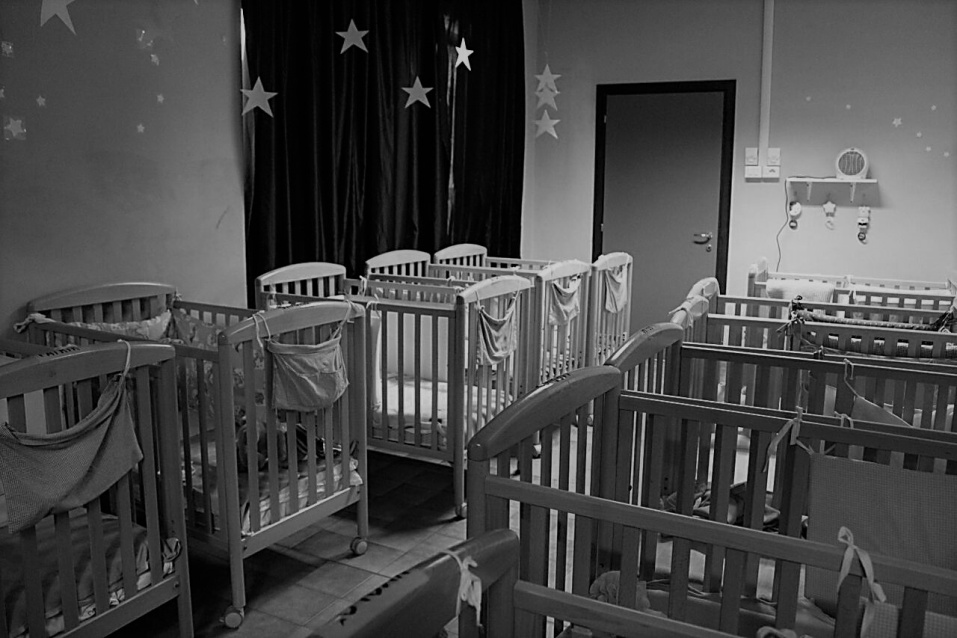


**S2 Fig D: furniture in preschool context (reading room)**


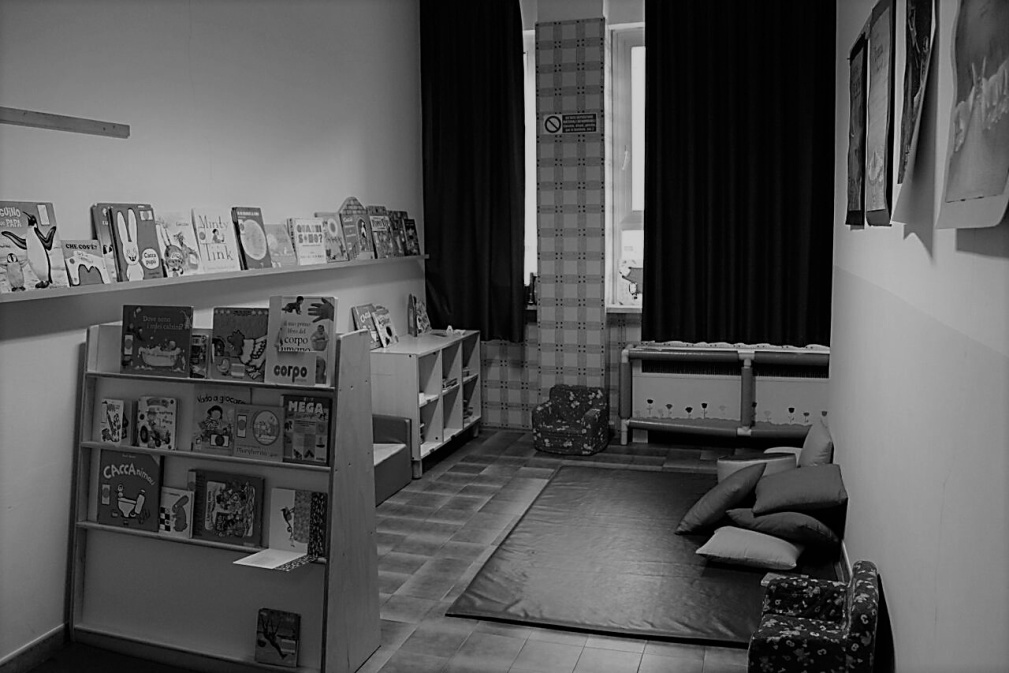


**S2 Fig E: Furnishing adopted following the participatory design**
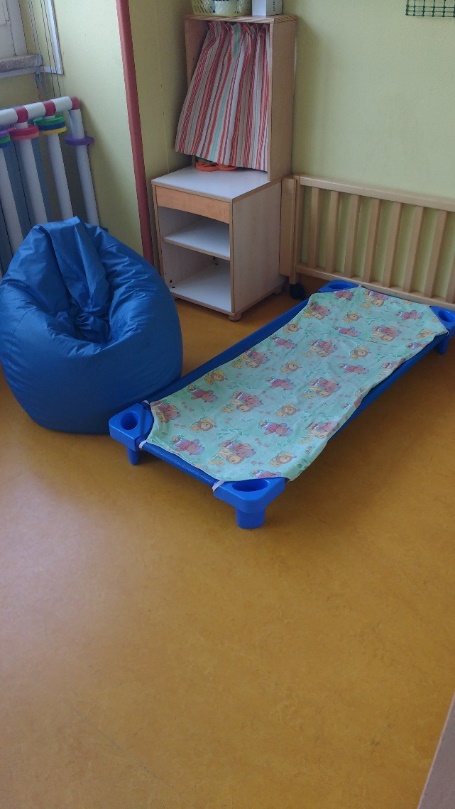


**S2 Fig F: Furnishing adopted following the participatory design**


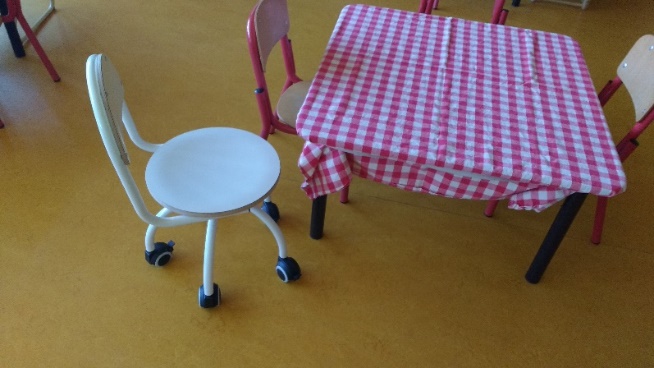

Supplement: S2 File — Harmful positions abstained by the preschool teachers and furniture in preschool context. (DOCX) [file pone.0206627.s002.docx]
